# Suppression of LFA-1 Expression by Spermine Is Associated with Enhanced Methylation of ITGAL, the LFA-1 Promoter Area

**DOI:** 10.1371/journal.pone.0056056

**Published:** 2013-02-13

**Authors:** Yoshihiko Kano, Kuniyasu Soda, Fumio Konishi

**Affiliations:** 1 Department of Surgery, Saitama Medical Center, Jichi Medical University, Saitama, Japan; 2 Department of Cardiovascular Research Institute, Saitama Medical Center, Jichi Medical University, Saitama, Japan; University of Pittsburgh, United States of America

## Abstract

Spermine and spermidine, natural polyamines, suppress lymphocyte function-associated antigen 1 (LFA-1) expression and its associated cellular functions through mechanisms that remain unknown. Inhibition of ornithine decarboxylase, which is required for polyamine synthesis, in Jurkat cells by 3 mM D,L-alpha-difluoromethylornithine hydrochloride (DFMO) significantly decreased spermine and spermidine concentrations and was associated with decreased DNA methyltransferase (Dnmt) activity, enhanced demethylation of the LFA-1 gene (ITGAL) promoter area, and increased CD11a expression. Supplementation with extracellular spermine (500 µM) of cells pretreated with DFMO significantly increased polyamine concentrations, increased Dnmt activity, enhanced methylation of the ITGAL promoter, and decreased CD11a expression. It has been shown that changes in intracellular polyamine concentrations affect activities of -adenosyl-L-methionine-decaroboxylase, and, as a result, affect concentrations of the methyl group donor, S-adenosylmethionine (SAM), and of the competitive Dnmt inhibitor, decarboxylated SAM. Additional treatments designed to increase the amount of SAM and decrease the amount of decarboxylated SAM–such as treatment with methylglyoxal bis-guanylhydrazone (an inhibitor of S-adenosyl-L-methionine-decaroboxylase) and SAM supplementation–successfully decreased CD11a expression. Western blot analyses revealed that neither DFMO nor spermine supplementation affected the amount of active Ras-proximate-1, a member of the Ras superfamily of small GTPases and a key protein for regulation of CD11a expression. The results of this study suggest that polyamine-induced suppression of LFA-1 expression occurs via enhanced methylation of ITGAL.

## Introduction

Lymphocyte function-associated antigen 1 (LFA-1), which consists of an alpha-L chain (CD11a) and a beta-2 chain (CD18), is one of the adhesion molecules expressed on cell membranes of a wide variety of leukocytes. LFA-1 binds intercellular adhesion molecules (ICAMs) expressed on endothelial cells. Binding of LFA-1 and ICAMs mediates activation and migration of leukocytes in immune and inflammatory processes [1]–[3]. Selective inhibition of LFA-1 function and, consequently, of immune function and inflammation can be achieved using a monoclonal antibody targeting LFA-1, or using a synthetic small molecule derived from compounds that mimic ICAM-1 [4]–[6].

Recent studies have shown that aging is accompanied by an enhanced pro-inflammatory status [7], [8]. Because chronic inflammation is thought to be involved in the pathogenesis of many, if not all, age-associated chronic diseases, increased pro-inflammatory status is thought to be one of the major factors that accelerate such diseases and aging itself. Among several predisposing factors favoring susceptibility to age-dependent chronic inflammatory diseases [9]–[11], an age-dependent increase in the proportion of cells expressing high levels of LFA-1 is one frequently documented in previous reports [11], [12]. The mechanisms of action of aging-associated changes in LFA-1 expression are not fully elucidated; however, mechanisms known to be involved in the regulation of LFA-1 expression are DNA methylation of the LFA-1 promoter area and intracellular signaling [13]–[15].

The natural polyamines spermidine and spermine are polycations found in every living cell in high micromolar to low millimolar concentrations. They are actively synthesized in rapidly growing cells, and synthesis decreases with aging. In addition to intracellular *de novo* synthesis, cells can acquire polyamines from the environment, e.g., from cells having increased polyamine synthesis and from the intestinal lumen. Polyamines are involved in signal transduction and gene expression [16]–[18], and their synthesis requires a methyl group donor, S-adenosylmethionine (SAM). We previously demonstrated that increases in extracellular spermine and spermidine suppress LFA-1 expression on non-stimulated human peripheral blood mononuclear cells (PBMCs) and also suppress cellular activities closely associated with known LFA-1 functions [19].

In this study, spermine was chosen to investigate the mechanism by which polyamines suppress LFA-1 expression, because the biological activity of spermine in this regard was more potent than that of spermidine *in vitro* [19], [20]. In addition, a significant inverse correlation was found between blood spermine concentration and LFA-1 expression on PBMCs from healthy volunteers, while that relationship regarding the spermidine concentration was tenuous [19].

## Materials and Methods

These study protocols involving human subjects were approved by the ethics committee of Saitama Medical Center, Jichi Medical University, Japan.

### Preparation of Human PBMCs

Human PBMCs were obtained from healthy volunteers. All 5 volunteers were members of our laboratory staff, including authors of this study. All participants provided their written informed consent to participate in this study and the ethics committees approved this consent procedure. They have to receive annual medical examinations, and exclusion criteria were as follows; regular use of any medicine, a history of hospital admission within the previous 6 months, a positive serological test for hepatitis B virus, human immunodeficiency virus, or hepatitis C virus, and any known health problem. Each volunteer donated 20 ml of peripheral blood 3 or 4 times using standard procedures, and PBMCs were isolated by density gradient centrifugation, using separate-L (Muto Pure Chemicals, Tokyo, Japan). Isolated PBMCs were washed 3 times in PBS(−), suspended in RPMI 1640 at 7×10^5^ cells/ml, and then supplemented with 10% heat-inactivated human serum AB (Wako Pure Chemicals, Osaka, Japan), 0.1% L-glutamine, and 0.01% penicillin-streptomycin (Invitrogen Life Technologies, CA, USA). Freshly prepared spermine (spermine tetrahydrochloride, Wako Pure Chemicals) was added the suspensions at various concentrations. All of the PBMCs were cultured for 72 h in culture plates and then harvested by gentle pipetting and 3 gentle PBS(−) washes before measurement of adhesion molecules.

### Preparation of Jurkat Cells

Jurkat cells (E6-1, ATCC Number: TIB-152), 3.0×10^5^ cells/ml, were cultured in RPMI-1640 supplemented with 10% heat-inactivated human serum (Cosmo Bio Co., Ltd. Tokyo, Japan) and 0.01% penicillin-streptomycin. Based on results of preliminary experiments done to determine concentrations that elicit significant biological activity without inducing cytotoxicity, the following reagents were added to cell cultures as appropriate at to achieve the following final concentrations: 3 mM D,L-alpha-difluoromethylornithine hydrochloride (DFMO) (ALEXIS Biochemicals Co., Lausen, Swirzerland); 500 µM spermine; 0.25 µM methylglyoxal bis-guanylhydrazone (MGBG) (Sigma-Aldrich Co., MO, USA); and 50 µM SAM (New England Biolabs Ltd., MA, USA). Cells were then cultured for 72 h, harvested and gently washed with PBS(−) 3 times for use in further experiments.

### Flow Cytometric Analysis

PBMCs and Jurkat cells cultured in a various conditions were fixed in 2% paraformaldehyde for 10 min at 4°C. To cells suspended in PBS(−) containing 0.1% bovine serum albumin (BSA) the following antibodies were added (5 µl per 5×10^5^ cells): fluorescein isothiocyanate (FITC)-conjugated anti-human CD11a, phycoerythrin (PE)-anti-CD11b, PE-anti-CD11c, FITC-anti-CD18, PE-anti-CD31, PE-anti-CD49d, PE-anti-CD49e, PE-anti-CD54, and FITC-ViaProbe (BD Pharmingen, NJ, USA). After incubating for 20 min at 4°C, cells were washed with 3 times PBS(−).

A FACScan flow cytometer (FACS Calibur, Becton, Dickinson and Company, NJ, USA) with CellQuest analysis software was used to identify 3–5×10^4^ human PBMCs gated in the lymphocyte and monocyte light-scattered regions and 1×10^6^ Jurkat cells gated in the lymphocyte light scatter region, which were then further analyzed.

### Measurement of Intracellular Polyamine Concentration in Jurkat Cells

Jurkat cells cultured for 72 h were harvested and washed 3 times in abundant PBS(−) to remove extracellular polyamines; 1×10^6^ cells were resuspended in 50 µl of 0.6 M perchloric acid and then were degraded by sonication (750 w, 30 sec) (Eyela Ultrasonic Cleaner, Tokyo Rikaikai Co., Tokyo, Japan) and vigorous vortexing. 300 µl dansyl chloride with 10 mg/ml acetone, 40 µM 1,7-diamninoheptane, and saturated sodium carbonate solution were added to the lysates. After incubation at 70°C for 15 min, 25 µl proline solution in water (100 mg/ml) and 500 µl toluene were added. The dried supernatant phases were dissolved in 500 µl acetonitrile. The concentrations of spermine and spermidine were measured by high-performance liquid chromatography (HPLC, LC-20AB, Shimdaz Corporation, Kyoto, Japan) with a Capcell pak C18 MG (Shiseido Co., Ltd., Tokyo, Japan).

### Bisulfite Sequencing of the LFA-1 Gene (ITGAL) Promoter in Jurkat Cells

Bisulfite sequencing was used to determine the methylation pattern of the ITGAL promoter (numbered relative to the transcription start site), as follows: 1–5 µg of purified DNA obtained from Jurkat cells cultured in various conditions was treated with 4 M sodium bisulfite and 0.2 M hydroquinone for 18 h. The 2.3-kb CD11a promoter fragments were amplified as 4 segments of sequential fragments using nested PCR. The primers were designed to account for the conversion of dC to dU by bisulfite and to avoid CG pairs. PCR conditions were as follows: 94°C for 2 min, and then 5 cycles of 94°C for 30 sec, 50–57°C for 30 sec, 72°C for 1 min; then 25 cycles of 94°C for 30 sec, 55–65°C for 30 sec, 72°C for 1 min; and then finally 10 min at 72°C. The annealing temperature was matched to the optimum conditions for each primer set. The Gene Amp PCR System 9700 (Applied Biosystems, CA, USA) was used for PCR. The primers used for primary and secondary PCR are shown in Tables 1 and 2.

**Table 1:** Primers used for bisulfite sequencing (primary PCR).

Segment 1	Forward:	GAAGAATTCTTTAGAAATTTAAGATTAGTTTGGGTAAGG
	Reverse:	CTCTCTAGATTTCTCCAATAACATATAAATAACCAACAT
Segment 2	Forward:	GAAGAATTCGTAGGTTGGTTTGAGTGTAGTGGTGTTTAAAAT
	Reverse:	CTCTCTAGAACTAAAACCACAAATACATACCACCATACCTAA
Segment 3	Forward:	TAGGAATTCTATATTTGTAATTTTAGTATTTTGGGAGGT
	Reverse:	GAATCTAGACAAAACAATATATTTAACCCATTTTAATTC
Segment 4	Forward:	AGGGAATTCTTAGGTTGGTTTTGATTTTTGGTTTTAAGAGAT
	Reverse:	CAATCTAGACTAAAACCAATCCCACTTTTAAAAAACAACTC

**Table 2:** Primers used for bisulfite sequencing (secondary PCR).

Segment 1	Forward:	CCGGAATTCAATTTAAGATTAGTTTGGGTAAGGTAGAGA
	Reverse:	CCCTCTAGAACCACAATAAAATACCCTTTACACCTACTT
Segment 2	Forward:	AAGGAATTCTTTTGTTTTAGTTTTTTAAGTAGTTGGGATTAT
	Reverse:	CCCTCTAGAATCTTAAACTCCTATACTCAAATAATCCTCCTA
Segment 3	Forward:	AGGGAATTCAGGAGTTTAAGATTAGTTTGGGTAATTAGT
	Reverse:	ATATCTAGAAAACTCACTAACTATAATTCCAACCCTTTA
Segment 4	Forward:	TGGGAATTCTGGATGTTAGTGAGAATTATGATAGTAGTG
	Reverse:	TTATCTAGATACAATTTCTCTAATCACAAACACATATCC

The amplified fragments were ligated into pGEM-T Easy Vector (Promega Co., WI, USA), and then cloned into ECOS competent *Escherichia coli* DH5 alpha (Nippon Gene Co., Ltd., Tokyo, Japan). The *E. coli* was cultured on Luria-Bertani nutrient agar containing 2% 5-bromo-4-chloro-3-indolyl-beta-D-galactoside (Takara Bio, Shiga, Japan) and 100 mM isopropyl-beta-D-thiogalactopyranoside (Takara Bio) for 24 h. Approximately 300 colonies were isolated and then cultured in Luria-Bertani medium for 9 h. The plasmid DNA was purified using a QuickLyse Miniprep Kit (Qiagen, Venlo, Netherlands), and then amplified with a BigDye Terminator v3.1 Cycle Sequencing Kit (Applied Biosystems) and T7 primer (GTAATACGACTCACTATAGGGC). Sequence reaction conditions were as follows: 96°C for 1 min, and 25 cycles of 96°C for 10 sec, 50°C for 5 sec and 60°C for 4 min. The reactive products were purified using a BigDye X Terminator purification kit (Applied Biosystems), and were analyzed using an ABI PRISM 3100 Genetic Analyzer (Applied Biosystems) and Data Collection Software ver.1.0.0.1 (Applied Biosystems).

### DNA Methyltrasferase (Dnmt) Activity Assay

EpiQuik Nuclear Extraction Kit I and EpiQuik DNA Methyltransferase Activity/Inhibition Assay Kits (Epigentek Group Inc., NY, USA) were used to determine Dnmt activity in the various experimental Jurkat cell cultures, according to the manufacturer’s protocols. Briefly, extracted nucleic acids from 1×10^7^ cells were incubated with kit assay buffer on cytosine-rich DNA-coated plates for 1 h. 5-methylcytosine antibody and color development solution were added to the plate, and absorbance was measured by a DTX 880 Multimode Detector (Beckman Coulter Inc., CA, USA); Multimode Analysis Software ver.3.2.0.5 (Beckman Coulter Inc.) was used for the analyses.

### Western Blotting for Active Ras-proximate-1 (Rap1)

The active Rap1 protein level in Jurkat cells was measured by western blotting. Active Rap1 Pull-Down and Detection Kits (Thermo Fisher Scientific Inc., MA, USA) were used for the experiments, according to the manufacturer’s protocol. Because Rap1 Pull-Down and Detection Kits is an immunoprecipitation based technique and does not provide any antibody against housekeeping protein, we have not detected any band like actin or beta microglobulin. Instead, a Pierce BCA Protein Assay Kit (Thermo Fisher Scientific Inc.) was used for the protein assay in order to ensure that the initial total protein content of each sample was identical. Extracted cell lysates containing proteins were then separated by gel electrophoresis using 4% T READY GELS J (Bio-Rad Laboratories, Inc., CA, USA) and 25 mM Tris, 192 mM glycine, 0.1% sodium dodecyl sulfate buffer, and then were transferred to Trans-Blot Transfer Medium Supported Nitrocellulose Membranes (Bio-Rad Laboratories, Inc.). The blotting buffer was 20% methanol in 25 mM Tris and 192 mM glycine. The membrane was blocked for 2 h with TBS (150 mM NaCl, 25 mM Tris) containing 3% BSA, and then washed with TBS containing 0.05% Tween-20 (TBST). The membrane was incubated in TBST containing 3% BSA, 0.1% sodium azide, and primary antibody at 4°C overnight. The primary antibody was anti-Rap1 antibody (1∶1000) (Thermo Fisher Scientific Inc.). The membrane was incubated for 1 h in TBST containing 7.5% non-fat dry milk and the secondary antibody, Pierce Goat Anti-Rabbit IgG (H+L), Peroxidase Conjugated (1∶20000) (Thermo Fisher Scientific Inc.). SuperSignal West Pico Chemiluminescent Substrate (Thermo Fisher Scientific Inc.) was used for exposing the membrane to X-ray film.

### Statistical Analysis

Data from analyses of flow cytometry and Dnmt assays were expressed as percentages compared to control cells (cells cultured with medium alone in each experiment). Data were expressed as means ± standard deviation of several experiments. Group means were compared using Student’s *t* tests; *p* values of less than 0.05 were considered statistically significant. The “jmp 6 Japanese edition” software (SAS Institute Inc., NC, USA) was used for all analyses.

## Results

### Spermine Suppressed the Mean Fluorescent Intensities (MFIs) of CD11a and CD18

**Figure 1:**
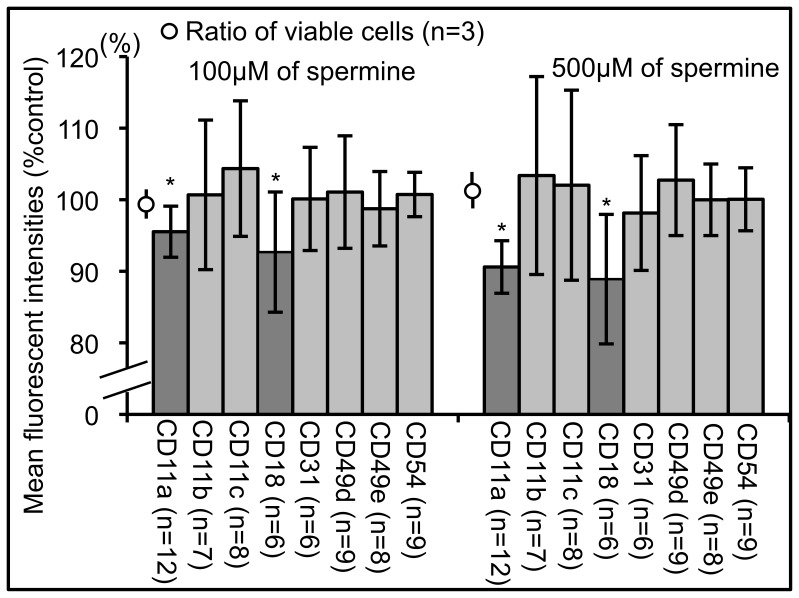
Effect of spermine on various membrane molecules expressed on human peripheral blood mononuclear cells. Expression of various adhesion molecules and Viaprobe on cells cultured for 72 h with spermine assessed by flow cytometry. Among membrane proteins, expression of lymphocyte function associated antigen 1 (CD11a and CD18) was suppressed. Means with standard bars, compared to cells cultured without spermine. *Significantly different from control cells. n = number of experiments.

Flow cytometric analysis revealed that spermine treatment for 72 h decreased the MFI of CD11a and CD18 staining on PBMCs gated in the lymphocyte and monocyte light-scattered regions, in a dose-dependent manner (Fig. 1). The suppression of CD11a and CD18 expression was not due to a decrease in the percentage of cells expressing CD11a (data not shown) [19] or to a cytotoxic effect of spermine, since the percentage of cells negative for ViaProbe was not changed (99.6% ±0.3%, n = 3, 100 µM; and 101.2% ±6.2%, n = 3, 500 µM). Although spermine decreased the MFIs of CD11a and CD18, it had no effect on the expression of CD11b, CD11c, CD31, CD49d, CD49e, or CD54.

### Intracellular Polyamine Concentration in Jurkat Cells

**Figure 2:**
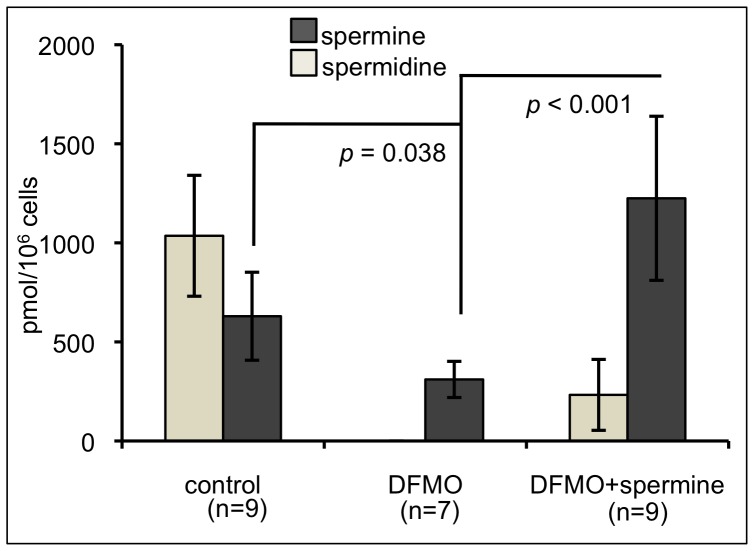
Changes in intracellular polyamine concentration in the presence of DFMO (an inhibitor of polyamine synthesis) and spermine supplementation. Polyamine concentrations in Jurkat cells cultured for 72 h in 3 conditions. Mean ± standard deviation. n = number of experiments. control: cells cultured in RPMI 1640 with 10% human serum. DFMO: cells treated with 3 mM DMFO. DFMO+spermine: cells treated with 3mM DFMO and 500 µM spermine. DFMO: D,L-alpha-difluoromethylornithine hydrochloride.

Jurkat cells cultured without treatment contained 629.64±222.09 pmol spermine/10^6^ cells and 1035.70±305.16 pmol spermidine/10^6^ cells. Intracellular concentrations of polyamines were decreased after 72 h treatment with DFMO, which inhibits ornithine decarboxylase (ODC), an enzyme required for polyamine synthesis. Intracellular spermidine was not detectable in cells after DFMO treatment, and spermine concentrations were decreased significantly, to 310.59±91.28 pmol/10^6^ cells (*p* = 0.038). When DFMO-supplemented Jurkat cells were treated with 500 µM spermine, the intracellular spermine concentrations increased significantly compared to cells treated with DFMO only, to 1224.88±414.12 pmol/10^6^ cells, which was higher than that of untreated control cells (*p*<0.001). Spermine supplementation also increased spermidine concentrations when compared to those of the cells cultured with DFMO (Fig. 2).

### Spermine, MGBG, and SAM Altered CD11a Expression

Flow cytometry revealed that polyamine depletion induced by 3 mM DFMO was associated with increased CD11a expression, to 111.38% ±3.94% (*p*<0.001) compared to control cells. Supplementation with 500 µM spermine of DFMO-treated cells decreased CD11a expression significantly, to 94.87% ±3.93% (*p*<0.001) compared to cells cultured with DFMO alone.

**Figure 3:**
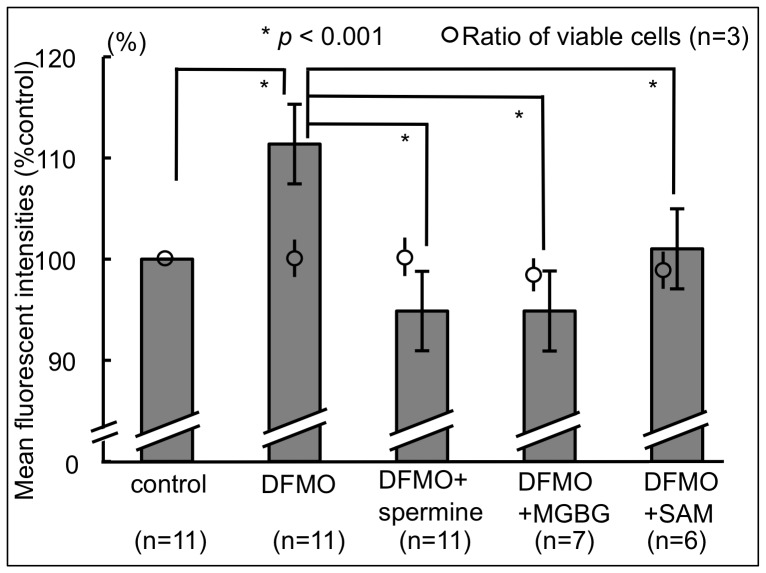
Effect of interventions affecting polyamine metabolism on CD11a expression. The mean fluorescent intensities of CD11a in Jurkat cells cultured for 72 h in various conditions were analyzed by flow cytometry. Mean ± standard deviation; n = number of experiments. control: cells cultured in unsupplemented culture medium. DFMO: cells cultured in control medium plus 3 mM DMFO. DFMO+spermine: cells cultured in control medium plus 3 mM DMFO and 500 µM spermine. DFMO+MGBG: cells cultured in control medium plus 3 mM DMFO and 0.25 µM MGBG. DFMO+SAM: cells cultured in control medium plus 3 mM DMFO and 50 µM SAM. DFMO: D,L-alpha-difluoromethylornithine hydrochloride, MGBG: methylglyoxal bis-guanylhydrazone, SAM: S-adenosylmethionine.

In order to test the hypothesis that changes in polyamine metabolism and resultant changes in the concentrations of decarboxylated SAM (dcSAM) and SAM affect the methylation status of ITGAL and induce the changes in LFA-1 expression, MGBG, an inhibitor of S-adenosyl-L-methionine-decarboxylase (AdoMetDC), was added to cells cultured with DFMO at a final concentration of 0.25 µM. MGBG treatment, which may result in an increased SAM and decreased dcSAM, significantly decreased CD11a expression, to 94.87% ±5.49%, compared to cells treated with DFMO alone (*p* = 0.007). Similarly, exogenous SAM supplementation of DFMO-treated Jurkat cells (to increase SAM availability) significantly decreased CD11a expression (*p*<0.001) (Fig. 3).

Again, ViaProbe assays revealed that the changes in CD11a expression were not due to changes in cell viability, because the ratio of cells negative for ViaProbe remained unchanged (DFMO, 100.9% ±1.96%, n = 3; DFMO+spermine, 99.99% ±2.02%, n = 3; DFMO+MGBG, 98.7% ±0.65%, n = 3; and DFMO+SAM, 99.1% ±1.27%, n = 3) (Fig. 3).

### Spermine Enhanced Jurkat Cell Dnmt Activity

**Figure 4:**
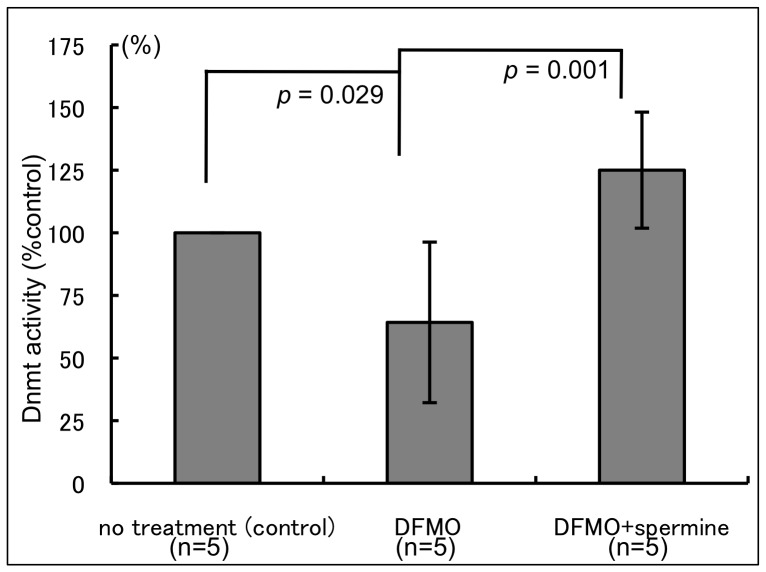
Effect of DFMO and spermine supplementation on Dnmt activity. DFMO decreased, while spermine supplementation increased Dnmt activity in Jurkat cells. Mean ±standard deviation; n = number of experiments as indicated. no treatment (control): cells cultured in culture medium. DFMO: cells cultured with 3 mM DMFO. DFMO+spermine: cells cultured with 3 mM DMFO and 500 µM spermine. DFMO: D,L-alpha-difluoromethylornithine hydrochloride.

DFMO treatment decreased Dnmt activity in Jurkat cells, when compared to that in cells cultured in medium supplemented with human serum (*p* = 0.029). Supplementation of DFMO-treated cells with 500 µM spermine significantly increased Dmnt activity (*p* = 0.001) (Fig. 4). The Dnmt activity changes were inversely related to changes in CD11a MFI.

### Spermine Affected the Methylation Status of the ITGAL Promoter

**Figure 5:**
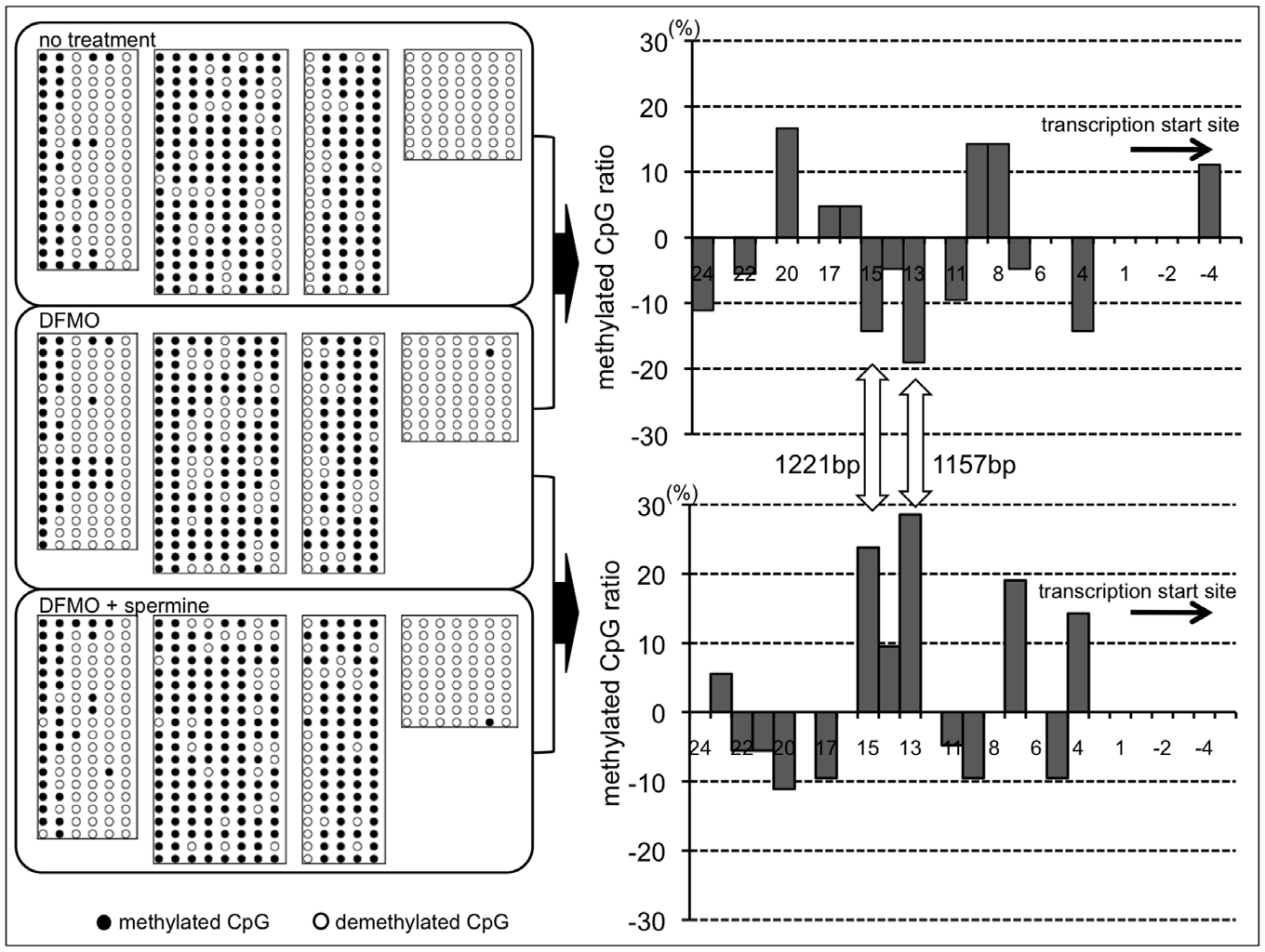
Changes in methylation status of ITGAL promoter by DFMO and spermine supplementation. Bisulfite sequencing was performed to determine the methylation pattern of the Jurkat cell ITGAL promoter, numbered relative to the transcription start site. Left: methylation status of each CpG dimer in 4 segments of sequential fragments. Each line: 1 experiment. Number of lines: number of experiments. Black circle: methylated CpG dimers. White circle: demethylated CpG dimers. Right upper: effects of DFMO on methylation status. Right lower: methylation status changes after spermine supplementation of cells treated with DFMO. Percentage: the increase of methylated CpG dimer; positive values, increased methylation; negative values, increased demethylation. ITGAL: LFA-1 gene, DFMO: D,L-alpha-difluoromethylornithine hydrochloride.

The methylation status of the ITGAL promoter area of Jurkat cells cultured in various treatment conditions (no treatment, 3 mM DFMO, and 3 mM DFMO supplemented with 500 µM spermine) was compared. There are 29 CpG dimers in the ITGAL promoter sequence that potentially can be methylated. The transcribed region was demethylated in all fragments irrespective of the conditions under which cells were cultured. The upper region of the ITGAL promoter, which contains *Alu* elements, was strongly methylated. Examination of approximately 300 colonies revealed that the change in methylation status was most significant at 1221 bp and 1157 bp. Polyamine depletion by DFMO enhanced demethylation of these areas, while spermine supplementation enhanced methylation, compared to cells treated with DFMO alone (Fig. 5).

### Western Blotting for Active Rap1

**Figure 6:**
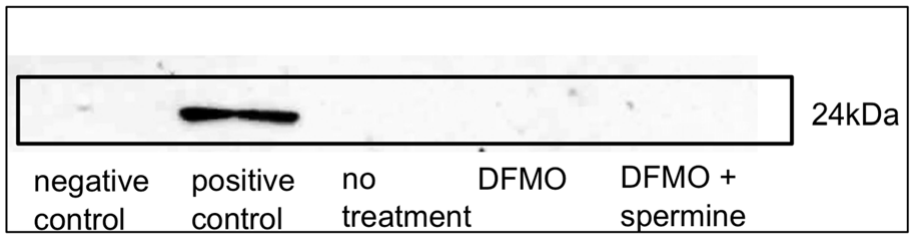
Effect of DFMO and spermine supplementation on Active Rap-1 protein levels. Neither DFMO nor spermine activated Rap-1. negative control: cell lysate treated with GDP. positive control: cell lysate treated with GTP-gamma-S. no treatment: cell lysate with no treatment. DFMO: lysate of cells cultured with 3 mM DMFO. DFMO+spermine: lysate of cells cultured with 3 mM DMFO and 500 µM spermine. Rap-1: Ras-proximate-1, DFMO: D,L-alpha-difluoromethylornithine hydrochloride.

Lysates of cells cultured in conditioned medium were used for negative and positive controls. Lysate treated with GTP gamma S to activate endogenous Rap1 had a strong band at 24 kDa, while no band was found in cell lysate treated with GDP (negaitive control). Western blotting for active Rap1 revealed that neither DFMO nor spermine supplementation activated Rap1 (Fig. 6).

## Discussion

Cytosine methylation of the CpG dinucleotide DNA sequence is associated with a variety of physiological phenomena [21]–[23]. In general, an increase in total DNA demethylation has been documented in aging salmon, mice, rats, cows and humans [24]–[26]. The age-dependent increase in LFA-1 expression is also considered to be due to age-dependent increase in demethylation of ITGAL, the LFA-1 promoter [13], [14]. Of interest is that polyamine synthesis decreases with aging because of age-dependent decreases in pertinent enzymatic activity [27], [28], and that suppression of an enzyme needed for polyamine synthesis, ODC, by ODC antizyme results in increased demethylation [29].

The CpG sites located at 1221 bp and 1157 bp in spermine-altered ITGAL have previously been shown to be associated with age-dependent increases in demethylation accompanied by increased LFA-1 expression [13], suggesting possible involvement of polyamine metabolism in aging-associated changes in DNA methylation. In general, methylation patterns are maintained during DNA replication [30]; however, *de novo* methylation and demethylation have been found in CpG dimers of several promoters [31], [32]. This study has shown that ITGAL is one of the promoters demonstrating CpG methylation status alteration by polyamine concentration change.

**Figure 7:**
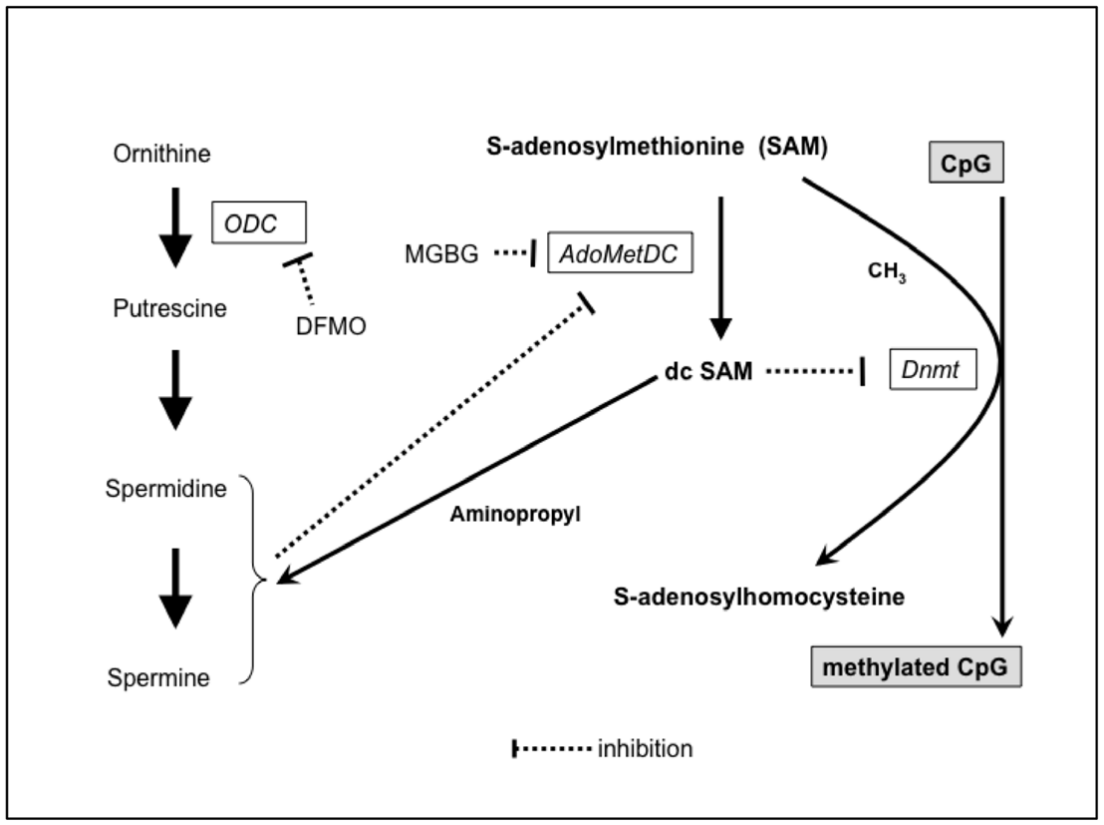
Relationship between polyamine metabolism and methylation. Increased dcSAM has been shown to inhibit Dnmt activity. The polyamines spermine and spermidine are synthesized from arginine and SAM. Inhibition of polyamine synthesis by DFMO increases dcSAM relative to SAM. Increased spermine from extracellular sources decreases conversion from SAM to dcSAM. Similarly, MGBG inhibits AdoMetDC activities, resulting in increased SAM and decreased dcSAM. SAM: S-adenosylmethionine, DFMO: D,L-alpha-difluoromethylornithine hydrochloride, dcSAM: decarboxylated S-adenosylmethionine, MGBG: methylglyoxal bis-guanylhydrazone, AdoMetDC: S-adenosyl-L-methionine-decarboxylase, ODC: ornithine decarboxylase. Dnmt: DNA methyltransferase.

The relationship between polyamine metabolism and DNA methylation is shown in Figure 7. SAM is a methyl group donor, and it is required for the synthesis of polyamine; dcSAM, derived from SAM, provides an aminopropyl molecule to putrescine and spermidine for synthesis of spermidine and spermine, respectively. Conversion from SAM to dcSAM is catalyzed by AdoMetDC. Changes in intracellular polyamines have been shown to affect the enzymatic activity of AdoMetDC, and, consequently, the concentrations of SAM and dcSAM; for example, a decrease in intracellular polyamines results in an increase of dcSAM relative to SAM [33]–[35], while an increase in intracellular polyamines inhibits AdoMetDC activity, resulting in an increase of SAM relative to dcSAM [34]–[37]. Increased availability of SAM enhances activity of the methylation catalyzing enzyme, Dnmt [38], [39], inhibiting regional hypomethylation and suppressing some genomes [40]. In contrast, an increase in dcSAM induced by inhibition of ODC activity has been shown to decrease Dnmt protein levels, inducing hypomethylation of the genome [29], [35]. Although we did not measure concentrations of SAM and dcSAM directly, the changes in Dnmt activity seen in this study were comparable to results of previous studies: polyamine depletion by DFMO decreased Dnmt activity and enhanced demethylation of ITGAL, and increased polyamine concentrations from extracellular sources enhanced both Dnmt activity and the ITGAL methylation status. In addition, the effects of the increased availability of SAM by MGBG treatment or SAM supplementation, although indirectly, further suggest that these mechanisms affect LFA-1 expression.

LFA-1 expression is also regulated by intracellular signaling pathways. Rap1, which is a 24 kDa G protein in the Ras superfamily, acts like a cellular switch and is vital for effective signal transduction [41]. Active Rap1 also regulates the expression of other adhesion molecules in the integrin family, such as very late antigen (VLA)-4 (CD49d/CD29) and VLA-5 (CD49e/CD29) [42], [43]. Therefore, the current results, showing that changes in spermine concentration did not affect Rap1 activation, and that suppression of expression was not observed for VLA-4 and -5, indicate that the role played by Rap-1 in polyamine-induced suppression of LFA-1 is minimal or nonexistent. Results of a recent study have demonstrated the involvement of histone acetylation in spermidine-induced gene expression [44], and histone acetylation has a close association with gene methylation [35], [45]. Considered together, these findings may suggest that a change in the methylation status of ITGAL, a promoter of LFA-1, is the main factor involved in polyamine-induced suppression of LFA-1 expression. While many adhesion molecules other than LFA-1 are not affected by polyamine supplementation, there must be other promoter areas like ITGAL that are affected by the changes in polyamine metabolism. It would be very interesting to find such genes; in addition, investigation on the effects of polyamine on the methylation status of the entire genome would be great interest.
